# Association between fish intake and prevalence of frailty in community-dwelling older adults after 4-year follow-up: the Korean frailty and aging cohort study

**DOI:** 10.3389/fnut.2023.1247594

**Published:** 2023-08-29

**Authors:** Jeonghwan Ahn, Miji Kim, Chang Won Won, Yongsoon Park

**Affiliations:** ^1^Department of Food and Nutrition, Hanyang University, Seoul, Republic of Korea; ^2^Department of Biomedical Science and Technology, College of Medicine, East-West Medical Research Institute, Kyung Hee University, Seoul, Republic of Korea; ^3^Department of Family Medicine, College of Medicine, Kyung Hee University, Seoul, Republic of Korea

**Keywords:** community-dwelling older adults, fish, frailty, n-3 polyunsaturated fatty acids, seafood

## Abstract

Cross-sectional epidemiological studies suggested the intake of fish and seafood was negatively associated with the prevalence of frailty. This study aimed to investigate the hypothesis that the prevalence of frailty is negatively associated with the consumption of total seafood and fish at baseline and 4-year follow-up. Using a multicenter longitudinal study of community-dwelling Korean adults aged 70–84 years old, 953 participants at baseline and 623 participants at 4-year follow-up were included after excluding participants without data on frailty or dietary intake in the Korean Frailty and Aging Cohort Study. Frailty was defined using the Cardiovascular Health Study index, and participants with scores ≥3 were considered frail. The trained dietitians obtained two non-consecutive 24-h dietary recalls during spring and fall at baseline. The prevalence of frailty was 13.5%. The intake of fish (OR 0.47; 95% CI 0.24–0.91; *p* for trend = 0.028) and total seafood (OR 0.34; 95% CI 0.18–0.68; *p* for trend = 0.002) at baseline was associated with frailty at 4-year follow-up after adjusting for the confounding factors. The intake of fish and total seafood at the baseline was negatively associated with the prevalence of exhaustion, low handgrip strength, and slow gait speed at 4-year follow-up. However, shellfish intake was not associated with frailty. In addition, the intake of fish, shellfish, and total seafood did not differ among the frailty transition groups in terms of deterioration, persistence, and reversal. The total consumption of seafood, particularly fish, could be beneficial for preventing frailty in Korean community-dwelling older adults. In particular, the consumption of fish (total seafood) at baseline could be beneficial for preventing exhaustion, low handgrip strength, and slow gait speed at 4-year follow-up.

## 1. Introduction

Frailty is a geriatric syndrome characterized by unintentional weight loss, exhaustion, low physical activity, low handgrip strength, and slow gait speed, and is related to adverse health outcomes such as falls, fractures, disability, hospitalization, and death (1). The older population is rapidly increasing worldwide, and the prevalence of frailty in older people has been estimated to be 12–24% (2).

The various risk factors associated with frailty include older age, sex, body mass index (BMI), and solitary life, polypharmacy, cognitive impairment, malnutrition, and dietary intake (3, 4). Vitamin D status, and consumption of protein, fruits, and vegetables have been suggested to be beneficial for preventing frailty (4). In addition, n-3 polyunsaturated fatty acids (PUFA), eicosapentaenoic acid (EPA), and docosahexaenoic (DHA), abundant in marine fish and shellfish, are well-known to possess anti-inflammatory properties, which may be related to the pathogenesis of frailty (4). Previously, we have showed that the erythrocyte level of n-3 PUFA, a biomarker of dietary intake of marine fish and shellfish, was inversely associated with the prevalence of frailty in older Koreans (5). Consistently, cross-sectional epidemiological studies have reported that frequent consumption of seafood or fish was inversely associated with the prevalence of frailty in older Japanese (6, 7). Fish intake was also negatively correlated with frailty score in older Ecuadorian (8) and the Irish (9). In addition, seafood consumption was positively associated with sufficient physical activity in older Croatians (10) and was positively associated with higher gait speed in older Norwegian women (11). The intake of oily fish was positively associated with handgrip strength in older UK adults (12, 13). Meta-analysis of clinical trials showed that supplementation of n-3 PUFA improved hand grip strength (14) and gait speed (15) in older Americans and Europeans.

Furthermore, Senior-ENRICA cohort studies have showed that ≥3 servings/week of fish or seafood, a component of the Mediterranean Diet Adherence Screener (MEDAS) (16), and ≥ 2 servings/week of seafood, a component of the Mediterranean Lifestyle Index (MEDLIFE) (17), were associated with reduced incidence of frailty during 2–4 years of follow-up. Additionally, higher than the median consumption of fish, a component of the Mediterranean Diet Score (MDS), was inversely associated with the incidence of frailty among older Spanish adults during the 2–4 year of follow-up (16).

Previous cross-sectional studies reported the association between the frailty and intake of fish and seafood as frequency but not amount, and two follow-up studies examined the association only among Spanish population. Compared to Spanish, Korean consumed more fatty fish and had higher levels do blood n-3 PUFA (18–21). Therefore, the present study aimed to investigate the hypothesis that the prevalence of frailty is negatively associated with the consumption of total seafood and fish at baseline and at 4-year follow-up in Korean community-dwelling older adults using data from the Korean Frailty and Aging Cohort Study (KFACS).

## 2. Methods

### 2.1. Participants

**Figure 1:**
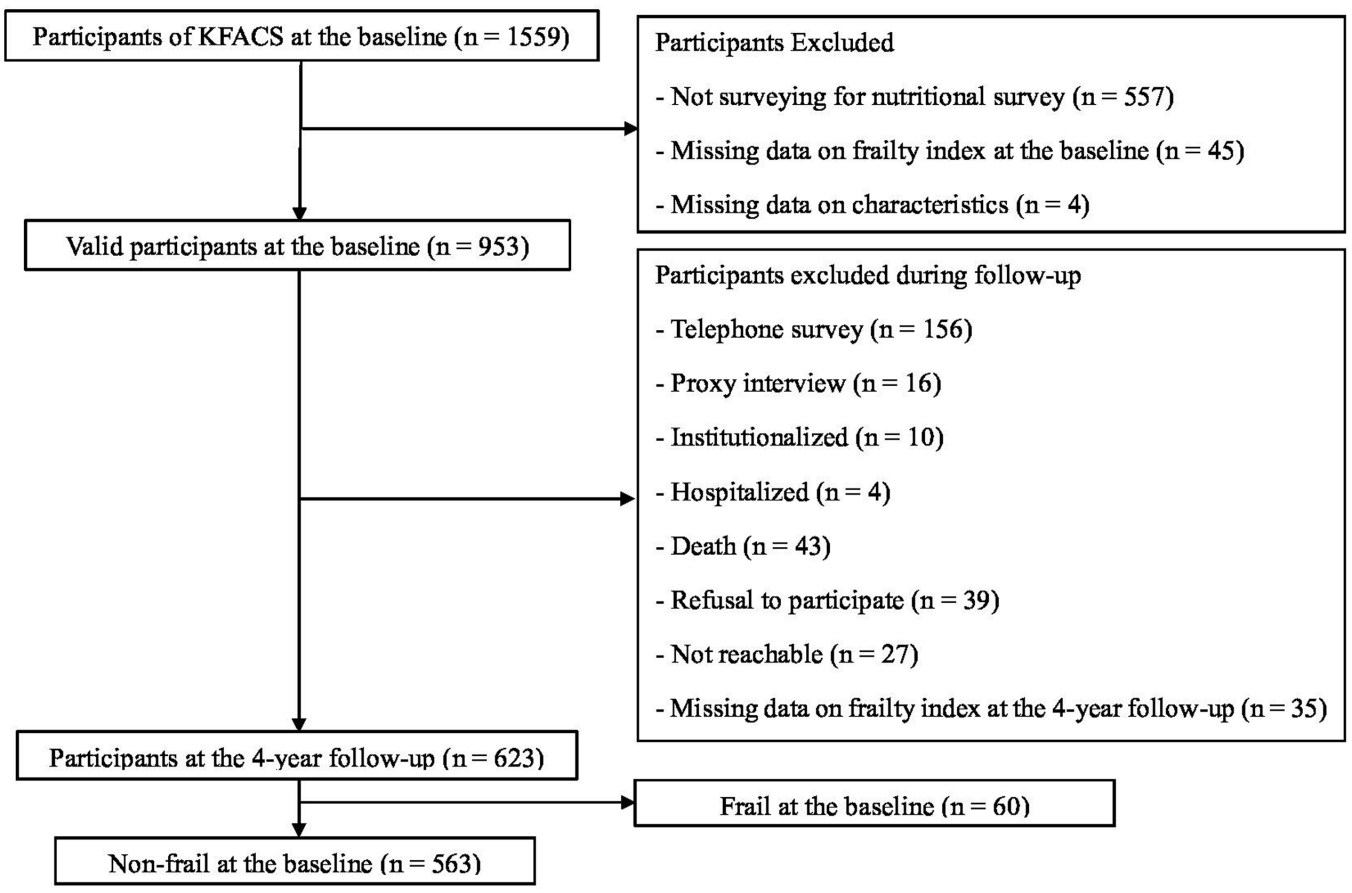
Flowchart of participant selection at the baseline and 4-year follow-up. KFACS, Korean Frailty and Aging Cohort Study.

Data from KFACS, a multicenter and longitudinal study of community-dwelling adults aged 70–84 years, was used (22). Sex- and age-stratified community residents drawn from 10 medical centers, including eight hospitals and two public health centers in urban and rural areas of Korea, were eligible for participation in the study. Of the 1,559 participants recruited from May 2016 to April 2017, 953 were included in the baseline analysis after excluding participants without data on dietary intake (*n* = 557), frailty index (*n* = 45), educational level (*n* = 2), and nutritional status (*n* = 2) (Figure 1). From among the 953 participants at baseline, 623 participants were included in the 4-year follow-up analysis for frailty prevalence after excluding participants who participated in a telephone survey (*n* = 156), proxy interview data of whom were available (*n* = 16), had died (*n* = 43), data on the frailty index of whom were missing (*n* = 35), were admitted to long-term care facilities or hospitals (*n* = 14), refused to participate (*n* = 39), and were not reachable (*n* = 27). For incident frailty at the 4-year follow-up, 563 participants who were not frail at baseline were included in the analysis. The KFACS protocol was approved by the Institutional Review Boards (KHUH-2015-12-103-107 and HYUIRB-202303-016). Written informed consent was obtained from all the participants.

### 2.2. Dietary intakes

Trained dietitians obtained dietary data using two non-consecutive 24-h dietary recalls during spring and fall at baseline. Bowls, plates, and food pictures developed by the National Institutes of Health (NIH) and Korea Disease Control and Prevention Agency (KDCA) were used to estimate the portion size. Intake of seafood was calculated using the dietary assessment system of the NIH and KDCA based on the National Rural Living Science Institute database (23). Total seafood included fish and shellfish. The fish included raw fish, canned fish, fish paste, and salted fish. Shellfish included clams, crabs, crayfish, lobsters, mussels, oysters, scallops, and shrimp.

### 2.3. Frailty assessment and transitions

The Cardiovascular Health Study (CHS) frailty index consists of five criteria: unintentional weight loss, exhaustion, low physical activity, low handgrip strength, and slow gait speed. Participants with scores ≥3 were considered frail (1). Unintentional weight loss was defined as ≥4.5 kg or 5% of the body weight loss during the previous year. Exhaustion was evaluated using the questions from the Center for Epidemiological Studies Depression (CES-D) scale and defined if the answer to either one of the questions, “I felt that everything I did was an effort” or “I could not get going,” was yes for three days or more a week (22). Low physical activity was calculated as the energy spent for a week by the International Physical Activity Questionnaire and defined as ≤494.65 kcal/week for men, and ≤ 283.50 kcal/week for women (22). Slow gait speed was defined as <1 m/s after walking 4 m, with 1.5 m before and after the walkway to allow for acceleration and deceleration (24). Low handgrip strength was measured twice for each hand using a digital hand grip dynamometer and defined as maximal grip strength <28 kg for men and < 18 kg for women (24). Transitions in frailty were divided into three groups according to changes in status from baseline to 4-year follow-up: deterioration (non-frail to pre-frail or frail, and pre-frail to frail), persistence (persistence of frail, pre-frail, or non-frail), and reversal (frail to pre-frail or non-frail, and pre-frail to non-frail).

### 2.4. Covariates

Information regarding age, sex, height, weight, living status, year of education (0–6, ≥7), economic status, smoking history (current, former, and never), history of falls during the last year, and prescribed medications during the past 3 months was collected. Cognitive impairment was defined when the Korean Mini-Mental State Examination score was less than 24 (22). Nutritional status was assessed using the Korean version of the Mini-Nutritional Assessment Short Form; score of 12–14 was defined to indicate normal nutritional status, 8–11 was associated with risk of malnutrition, and < 7 with malnutrition (22). Comorbid status was determined by the presence of 0, 1, and ≥ 2 of the following diseases: hypertension, diabetes mellitus, cancer, chronic obstructive pulmonary disease, myocardial infarction, heart failure, angina, asthma, arthritis, cerebral ischemic, or renal disease. Weight was measured to the nearest 0.1 kg using a portable digital scale, and height was measured to the nearest 0.1 cm using a measuring tape.

### 2.5. Statistical analyses

Statistical analyses were performed using the SPSS software (version 27.0; SPSS Inc., Chicago, IL, United States). The Kolmogorov–Smirnov test was used to check the normal distribution of the variables. Continuous variables were presented as mean ± standard deviation (SD) and analyzed using the independent *t*-test for parametric variables and the Mann–Whitney test for non-parametric variables. The proportions of categorical variables were presented as the number of participants using the chi-squared test. In the multivariate models, covariates with value of *p* < 0.20 were selected as confounding factors and included in the fully adjusted model (25). The confounding factors, namely, age, sex, medications, cognitive impairment, falls, and nutritional status, were included in the fully adjusted model. Odds ratios (ORs) and 95% confidence intervals (CIs) were used to determine the associations between total seafood, fish, and shellfish intake and frailty and each frailty criterion using multivariate logistic regression analysis. The lowest tertile of intake of total seafood, fish, and shellfish was considered the reference group, and the value of ps for trend were calculated using the median value of each tertile. The intake of total seafood, fish, and shellfish was compared among the three frailty transition groups (deterioration, persistence, and reversal) using analysis of covariance (ANCOVA) after adjusting for covariates.

## 3. Results

### 3.1. Characteristics of participants

Prevalence of frailty was 12.3% at the baseline and 13.5% at the 4-year follow-up, and prevalence of all the five frailty criteria was higher in frail than in non-frail participants (Table 1). At the baseline and 4-year follow-up, the frail participants were older, had higher proportion of women, non-smokers, cognitive impairment, medications, malnutrition, and falls in the last year, and lower education than the non-frail participants. The frail participants showed a lower proportion of living alone than the non-frail participants at baseline and had lower BMI than the non-frail participants at the 4-year follow-up. The intake of total seafood, fish, and shellfish was significantly lower in the frail than in the non-frail participants at baseline and at the 4-year follow-up.

**Table 1:** Characteristics of the participants with and without frailty at the baseline and 4-year follow-up.

	Baseline			Follow-up		
	Non-frail (*n* = 836)	Frail (*n* = 117)	*p-*value	Non-frail (*n* = 539)	Frail (*n* = 84)	*p-*value
Frailty score^a^	0.77 ± 0.77	3.22 ± 0.48	<0.001	0.71 ± 0.77	3.33 ± 0.55	<0.001
Frailty criteria, n (%)						
Unintentional weight loss	41 (4.9)	33 (28.2)	<0.001	24 (4.5)	31 (36.9)	<0.001
Exhaustion	218 (26.1)	97 (82.9)	<0.001	123 (22.8)	73 (86.9)	<0.001
Low physical activity	63 (7.5)	49 (41.9)	<0.001	27 (5.0)	36 (42.9)	<0.001
Low handgrip strength	169 (20.2)	93 (79.5)	<0.001	100 (18.6)	67 (79.8)	<0.001
Slow gait speed	149 (17.8)	105 (89.7)	<0.001	110 (20.4)	73 (86.9)	<0.001
Age (years)	75.92 ± 3.81	78.79 ± 3.85	<0.001	79.47 ± 3.71	82.45 ± 3.65	<0.001
Women, *n* (%)	414 (49.5)	77 (65.8)	0.001	269 (49.9)	52 (61.9)	0.046
Body mass index (kg/m^2^)	24.43 ± 2.84	24.37 ± 3.68	0.859	24.41 ± 2.86	23.22 ± 4.11	0.012
Smoking, *n* (%)			0.006			0.034
Never	494 (59.1)	80 (68.4)		301 (56.0)	57 (67.8)	
Former	305 (36.5)	27 (23.1)		213 (39.7)	24 (28.6)	
Current	37 (4.4)	10 (8.5)		23 (4.3)	3 (3.6)	
Education, *n* (%)			<0.001			<0.001
0–6	346 (41.4)	89 (76.1)		214 (39.7)	56 (66.7)	
≥7	490 (58.6)	28 (23.9)		325 (60.3)	28 (33.3)	
Living alone, *n* (%)	205 (24.5)	38 (32.5)	0.039	141 (26.2)	27 (32.1)	0.570
Comorbidity, *n* (%)^b^			0.001			0.027
0	193 (23.1)	14 (12.0)		118 (21.9)	11 (13.1)	
1	296 (35.4)	32 (27.4)		176 (32.7)	22 (26.2)	
≥2	347 (41.5)	71 (60.7)		245 (45.5)	51 (60.7)	
Number of medications	4.25 ± 7.96	5.85 ± 9.23	0.001	4.56 ± 5.23	5.67 ± 3.33	0.001
Cognitive impairment, *n* (%)^c^	155 (18.5)	55 (47.0)	<0.001	96 (17.8)	44 (52.4)	<0.001
Fall in the last year, *n* (%)	162 (19.4)	36 (30.8)	0.007	120 (22.3)	36 (42.9)	<0.001
Nutritional status, *n* (%)			<0.001			<0.001
Normal	737 (88.2)	78 (66.7)		437 (81.2)	42 (50.0)	
At risk of malnutrition	97 (11.6)	35 (29.9)		95 (17.7)	30 (35.7)	
Malnutrition	2 (0.2)	4 (3.4)		6 (1.1)	12 (14.3)	
Total seafood (g/day)	51.43 ± 56.47	25.82 ± 32.35	<0.001	53.67 ± 54.33	30.98 ± 44.05	<0.001
Fish (g/day)	39.35 ± 45.18	21.09 ± 28.89	<0.001	40.95 ± 44.78	24.75 ± 33.71	<0.001
Shellfish (g/day)	12.08 ± 30.26	4.73 ± 10.68	0.009	12.71 ± 27.58	6.22 ± 15.48	0.036

Data were presented as mean ± SD or number of the participants (percentage distribution), as appropriate. Value of ps were analyzed using the independent *t*-test for parametric continuous variables and Mann–Whitney for non-parametric continuous variables and chi-squared test for categorical variables.

a Frailty score was assessed using the Cardiovascular Health Study frailty index, which consists of five criteria: unintentional weight loss, exhaustion, low physical activity, low handgrip strength, and slow gait speed.

b Comorbid status was determined by the presence of 0, 1, and ≥ 2 of the following diseases: hypertension, diabetes mellitus, cancer, chronic obstructive pulmonary disease, myocardial infarction, heart failure, angina, asthma, arthritis, cerebral ischemic, or renal disease.

c Cognitive impairment was assessed using a Korean Mini-Mental State Examination score of less than 24.

### 3.2. Association between frailty and intake of seafood

In the multivariable-adjusted model, the intake of total seafood and fish at baseline was inversely associated with the prevalence of frailty at baseline and at the 4-year follow-up (Table 2). Additionally, a negative association was observed between the prevalence of frailty and intake of total seafood and fish as continuous variables at baseline and 4-year follow-up. After adjusting for confounding factors, the prevalence of frailty was not associated with shellfish intake as a continuous or non-continuous variable at baseline or 4-year follow-up. Although the intake of fish, total seafood, and shellfish tended to be higher in the persistent or reversed frailty transition groups than in the deterioration group, there was no significant difference between the frailty transition groups after adjusting for confounding factors (Table 3). In addition, the incidence of frailty was not associated with the intake of total seafood, fish, or shellfish after adjusting for confounding factors (Supplementary Table S1).

**Table 2:** Logistic regression of seafood intake for the prevalence of frailty at the baseline and 4-year follow-up.

	Tertiles of dietary intake			*p* for trend	Dietary intake continuous	
	T1	T2	T3		OR (95% CI)	*p*-value
Baseline (n = 953)
Total seafood, g/day	≤ 12.00	12.00 < to ≤55.70	> 55.70			
Frail, *n* (%)	67 (20.9)	30 (9.6)	20 (6.3)			
Adjusted OR (95% CI)	1.0 (ref)	0.448 (0.271–0.742)	0.336 (0.188–0.600)	<0.001	0.988 (0.982–0.995)	<0.001
Fish, g/day	≤ 7.50	7.50 < to ≤41.50	> 41.50			
Frail, *n* (%)	60 (18.3)	34 (11.0)	23 (7.3)			
Adjusted OR (95% CI)	1.0 (ref)	0.548 (0.332–0.904)	0.453 (0.262–0.785)	0.008	0.989 (0.982–0.996)	0.002
Shellfish, g/day	0	0 < to ≤5.00	> 5.00			
Frail, *n* (%)	70 (13.2)	22 (18.8)	25 (8.14)			
Adjusted OR (95% CI)	1.0 (ref)	1.821 (1.007–3.293)	0.804 (0.476–1.360)	0.282	0.984 (0.967–1.001)	0.064
Follow-up (*n* = 623)						
Total seafood, g/day	≤ 16.88	16.88 < to ≤57.25	> 57.25			
Frail, *n* (%)	46 (22.2)	23 (11.0)	15 (7.2)			
Adjusted OR (95% CI)	1.0 (ref)	0.514 (0.281–0.941)	0.344 (0.175–0.676)	0.002	0.991 (0.985–0.998)	0.009
Fish, g/day	≤ 8.25	8.25 < to ≤42.50	> 42.50			
Frail, *n* (%)	40 (19.2)	28 (13.4)	16 (7.8)			
Adjusted OR (95% CI)	1.0 (ref)	0.710 (0.395–1.277)	0.467 (0.240–0.911)	0.028	0.991 (0.984–0.999)	0.020
Shellfish, g/day	0	0 < to ≤6.00	> 6.00			
Frail, *n* (%)	46 (13.9)	17 (20.5)	21 (10.1)			
Adjusted OR (95% CI)	1.0 (ref)	1.872 (0.930–3.768)	0.975 (0.531–1.791)	0.752	0.990 (975–1.005)	0.199

OR, odds ratio; CI, confidence interval; ref.; reference. Estimate of p for linear trends was based on linear scores derived from the medians of tertiles of seafood intake among all participants. Adjusted OR and 95% CI were analyzed using logistic regression analysis after adjusting for sex, age, medications, cognitive impairment, fall experience, and nutritional status. Estimates of value of ps were derived from the continuous variables of dietary intake among all participants after adjusting for sex, age, medications, cognitive impairment, fall experience, and nutritional status.

**Table 3:** Intake of seafood according to the transition of frailty.

	Transition of frailty (*n* = 623)^a^			Unadjusted *p*-value	Adjusted *p*-value
(g/day)	Deterioration (*n* = 155)	Persistence (*n* = 334)	Reversal (*n* = 134)		
Total seafood	45.14 ± 54.13	51.26 ± 51.30	55.31 ± 58.24	0.260	0.420
Fish	34.88 ± 44.06	38.57 ± 41.87	43.79 ± 47.82	0.225	0.256
Shellfish	10.26 ± 20.64	12.70 ± 29.53	11.52 ± 23.85	0.628	0.763

*p*-value was determined using ANCOVA for normally distributed continuous variables of seafood intake after adjusting for age, body mass index, smoking, and nutritional status.

a Transitions of frailty were divided into three groups according to changes in status from baseline to the 4-year follow-up: deterioration (non-frail to pre-frail or frail, and pre-frail to frail), persistence (persistence of frail, pre-frail, or non-frail), and reversal (frail to pre-frail or non-frail, and pre-frail to non-frail).

### 3.3. Association between the five frailty criteria and intake of seafood

Regarding each frailty criterion, multivariate-adjusted logistic regression analysis showed that the intake of total seafood and fish was inversely associated with the prevalence of exhaustion, and that the intake of total seafood was inversely associated with the prevalence of slow gait speed after adjusting for confounding factors at baseline (Table 4). At baseline, the middle tertile of total seafood and fish intake was inversely associated with the prevalence of low handgrip strength compared with the lowest tertile after adjusting for confounding factors. In addition, the total seafood and fish intake was negatively associated with the prevalence of exhaustion, low handgrip strength, and slow gait speed at the 4-year follow-up after adjusting for confounding factors. Association between shellfish intake and each frailty criterion at baseline or at the 4-year follow-up was not observed after adjusting for confounding factors.

**Table 4:** Logistics regression of seafood intake for each frailty criterion at the baseline and 4-year follow-up.

	Tertiles of dietary intake (g/day)			*p* for trend
	T1	T2	T3	
Baseline (n = 953)				
Total seafood, g/day	≤ 12.00	12.00 < to ≤55.70	> 55.70	
Weight loss, *n* (%)	27 (8.4)	25 (8.0)	22 (6.9)	
Adjusted OR (95% CI)	1.0 (ref)	1.132 (0.617–2.076)	0.808 (0.425–1.537)	0.454
Exhaustion, *n* (%)	138 (43.0)	105 (33.4)	72 (22.6)	
Adjusted OR (95% CI)	1.0 (ref)	0.705 (0.502–0.989)	0.480 (0.333–0.691)	0.001
Low physical activity, *n* (%)	50 (15.6)	33 (10.5)	29 (9.12)	
Adjusted OR (95% CI)	1.0 (ref)	0.709 (0.436–1.154)	0.592 (0.352–1.040)	0.082
Low handgrip strength, *n* (%)	106 (33.0)	71 (22.6)	85 (26.7)	
Adjusted OR (95% CI)	1.0 (ref)	0.680 (0.470–0.984)	0.913 (0.631–1.322)	0.843
Slow gait speed, *n* (%)	122 (38.0)	74 (23.6)	58 (18.2)	
Adjusted OR (95% CI)	1.0 (ref)	0.564 (0.390–0.816)	0.509 (0.343–0.754)	0.001
Fish, g/day	≤ 7.50	7.50 < to ≤41.50	> 41.50	
Weight loss, *n* (%)	26 (7.9)	25 (8.1)	23 (7.3)	
Adjusted OR (95% CI)	1.0 (ref)	1.059 (0.577–1.944)	0.964 (0.514–1.808)	0.863
Exhaustion, *n* (%)	134 (40.9)	102 (33.1)	79 (25.0)	
Adjusted OR (95% CI)	1.0 (ref)	0.703 (0.500–0.998)	0.573 (0.402–0.816)	0.004
Low physical activity, *n* (%)	46 (14.0)	37 (12.0)	29 (9.1)	
Adjusted OR (95% CI)	1.0 (ref)	0.896 (0.555–1.448)	0.701 (0.420–1.170)	0.173
Low handgrip strength, *n* (%)	106 (32.3)	71 (23.1)	85 (26.8)	
Adjusted OR (95% CI)	1.0 (ref)	0.687 (0.474–0.996)	0.943 (0.657–1.356)	0.624
Slow gait speed, *n* (%)	111 (33.8)	72 (23.4)	71 (22.4)	
Adjusted OR (95% CI)	1.0 (ref)	0.623 (0.427–0.908)	0.748 (0.512–1.093)	0.279
Shellfish, g/day	0	0 < to ≤5.0	> 5.00	
Weight loss, *n* (%)	40 (7.6)	11 (9.4)	23 (7.5)	
Adjusted OR (95% CI)	1.0 (ref)	0.980 (0.461–2.086)	1.003 (0.564–1.785)	0.983
Exhaustion, *n* (%)	188 (35.5)	43 (36.8)	84 (27.4)	
Adjusted OR (95% CI)	1.0 (ref)	1.100 (0.711–1.701)	0.763 (0.551–1.057)	0.081
Low physical activity, *n* (%)	73 (13.8)	16 (13.7)	23 (7.5)	
Adjusted OR (95% CI)	1.0 (ref)	1.043 (0.572–1.901)	0.637 (0.368–1.050)	0.069
Low handgrip strength, *n* (%)	153 (28.9)	38 (32.5)	71 (23.1)	
Adjusted OR (95% CI)	1.0 (ref)	1.305 (0.825–2.063)	0.927 (0.657–1.308)	0.538
Slow gait speed, *n* (%)	163 (30.8)	34 (29.1)	57 (18.6)	
Adjusted OR (95% CI)	1.0 (ref)	1.067 (0.656–1.734)	0.724 (0.498–1.054)	0.079
Follow-up (*n* = 623)				
Total seafood, g/day	≤ 16.88	16.88 < to ≤57.25	> 57.25	
Weight loss, *n* (%)	21 (10.1)	18 (8.6)	16 (7.7)	
Adjusted OR (95% CI)	1.0 (ref)	0.943 (0.474–1.877)	0.762 (0.374–1.552)	0.443
Exhaustion, *n* (%)	84 (40.5)	64 (30.6)	48 (23.2)	
Adjusted OR (95% CI)	1.0 (ref)	0.708 (0.458–1.093)	0.584 (0.369–0.924)	0.027
Low physical activity, *n* (%)	25 (12.1)	21 (10.0)	17 (8.2)	
Adjusted OR (95% CI)	1.0 (ref)	0.912 (0.484–1.720)	0.769 (0.390–1.514)	0.446
Low handgrip strength, *n* (%)	79 (38.2)	46 (22.0)	42 (20.3)	
Adjusted OR (95% CI)	1.0 (ref)	0.529 (0.334–0.839)	0.492 (0.306–0.791)	0.006
Slow gait speed, *n* (%)	84 (40.6)	51 (24.4)	48 (23.2)	
Adjusted OR (95% CI)	1.0 (ref)	0.554 (0.348–0.884)	0.542 (0.336–0.874)	0.020
Fish, g/day	≤ 8.25	8.25 < to ≤42.50	> 42.50	
Weight loss, *n* (%)	17 (8.2)	19 (9.1)	19 (9.2)	
Adjusted OR (95% CI)	1.0 (ref)	1.181 (0.582–2.397)	1.206 (0.591–2.461)	0.653
Exhaustion, *n* (%)	79 (38.0)	71 (34.0)	46 (22.3)	
Adjusted OR (95% CI)	1.0 (ref)	0.887 (0.577–1.365)	0.599 (0.378–0.950)	0.025
Low physical activity, *n* (%)	23 (11.1)	21 (10.0)	19 (9.2)	
Adjusted OR (95% CI)	1.0 (ref)	0.973 (0.512–1.850)	0.984 (0.506–1.915)	0.973
Low handgrip strength, *n* (%)	68 (32.7)	55 (26.3)	44 (21.4)	
Adjusted OR (95% CI)	1.0 (ref)	0.791 (0.503–1.244)	0.611 (0.379–0.983)	0.048
Slow gait speed, *n* (%)	83 (39.9)	52 (25.0)	48 (23.3)	
Adjusted OR (95% CI)	1.0 (ref)	0.532 (0.333–0.850)	0.566 (0.353–0.909)	0.043
Shellfish, g/day	0	0 < to ≤6.00	> 6.00	
Weight loss, *n* (%)	32 (9.6)	10 (12.0)	13 (6.3)	
Adjusted OR (95% CI)	1.0 (ref)	1.262 (0.552–2.676)	0.680 (0.342–1.352)	0.234
Exhaustion, *n* (%)	109 (32.8)	29 (34.9)	58 (27.9)	
Adjusted OR (95% CI)	1.0 (ref)	1.093 (0.634–1.884)	0.914 (0.607–1.376)	0.619
Low physical activity, *n* (%)	32 (9.6)	12 (14.5)	19 (9.1)	
Adjusted OR (95% CI)	1.0 (ref)	0.737 (0.455–1.195)	0.601 (0.358–1.010)	0.984
Low handgrip strength, *n* (%)	99 (29.8)	26 (31.3)	42 (20.2)	
Adjusted OR (95% CI)	1.0 (ref)	1.189 (0.675–2.095)	0.736 (0.475–1.141)	0.136
Slow gait speed, *n* (%)	108 (32.5)	25 (30.1)	50 (24.0)	
Adjusted OR (95% CI)	1.0 (ref)	0.917 (0.508–1.655)	0.844 (0.547–1.303)	0.456

OR, odds ratio; CI, confidence interval; ref.; reference. Estimate of *p*-values for linear trends was based on linear scores derived from the medians of tertiles of seafood intake among all participants. Adjusted OR and 95% CI were analyzed using logistic regression analysis after adjusting for sex, age, medications, cognitive impairment, fall experience, and nutritional status.

## 4. Discussion

The present study showed that the intake of total seafood and fish at baseline was inversely associated with the prevalence of frailty at baseline and at the 4-year follow-up among community-dwelling Koreans aged 70–84 years. Consistent with the observations of the present study, previous cross-sectional epidemiological studies have reported beneficial effect of seafood or fish intake measured by frequency on frailty (6, 7, 26). Yamaguchi et al. (6) showed that the prevalence of frailty was inversely associated with the intake of seafood in older Japanese women, and was significantly lower from the second quartile, with ≥7.7 servings/week of seafood. The prevalence of frailty was also significantly lower in older Japanese women with rheumatoid arthritis who had ≥3 servings/week of fish (26) and in older Japanese women with ≥7 servings/week of fish (7). In addition, frailty score was negatively correlated with the intake of fish in older Ecuadorian (8) and the Irish (9). Moradell et al. (27) reported that intake of n-3 PUFA has been shown to be significantly lower in frail (2.0 ± 0.2 g/day) than in non-frail (3.2 ± 0.3 g/day) older Spanish adults. Previously, we have shown that the erythrocyte levels of n-3 PUFA, such as EPA and DHA were inversely associated with the likelihood of frailty in older Korean adults from KFACS (22). In the secondary analysis of the Multidomain Alzheimer Preventive Trial (MAPT) study, erythrocyte levels of EPA and DHA were lower in frail than in non-frail older French adults with spontaneous memory complaint, limitations in one instrumental activity of daily living, or < 0.8 m/s gait speed at the baseline (28). However, in the MAPT study, the erythrocyte levels of EPA and DHA were not significantly associated with the incidence of frailty in the 5-year follow-up, as 50% participants were supplemented with 1 g/day of EPA and DHA during the first 3 years (28).

During the 2–4 year follow-up, the Senior ENRICA cohort study showed that higher than the median intake of fish consumption as a component of the MDS, was associated with reduced incidence of frailty in older Spanish adults (16). Similarly, the present study showed that the intake of total seafood and fish was significantly higher in non-frail participants than in frail participants. Additionally, the Senior ENRICA cohort studies showed that consumption of ≥3 servings/week of fish (≥300 g/week) or seafood (≥600 g/week) as a component of the MEDAS (16), and ≥ 2 servings/week of seafood (≥200–300 g/week) as a component of the MEDLIFE (17), was associated with reduced incidence of frailty in older Spanish adults during 2–4 years of follow-up. Previous studies have compared the risk of frailty with ≥2–3 servings/week of seafood, a component of the MEDAS (16) or MEDLIFE (17). However, this is the first study to show that the intake of not only total seafood, but also of fish, was negatively associated with the prevalence of frailty during the 4-year follow-up. In the present study, the prevalence of frailty was significantly lower when ≥297 g fish /week was consumed, similar to that observed in the Senior ENRICA cohort study where 300 g fish was consumed weekly (16). Furthermore, the prevalence of frailty was significantly lower when >118 g total seafood /week was consumed in the present study, which was lower than the seafood consumption of ≥200 g/week in the Senior ENRICA cohort study (17). Schuchardt et al. (20). showed that the erythrocyte levels of EPA and DHA were 9.26% in Koreans and 7.05% in the Spanish. Consistently, the Korean National Health and Nutrition Survey reported that Korean older adults consumed 1.59 g/day of n-3 PUFA (29), which was higher than that consumed by Spanish older adults (1.1 g/day of n-3 PUFA) according to the Anthropometry, Intake, and Energy Balance study in Spain (30). In addition, consumption of fatty fish was approximately 54% of the total fish consumed in the present study and 36% of the total fish consumed in the Senior-ENRICA cohort study (19).

The Osteoarthritis Initiative (OAI) multicenter study showed that ≥13 servings of fish/month as a component of the Mediterranean diet score was not associated with the incidence of frailty among patients at high risk or having knee osteoarthritis during an 8-year follow-up (31), unlike the present study used the CHS frailty index, the OAI study used the Study of Osteoporotic Fractures (SOF) index, which has been shown not to be associated with mortality, functional decline, and hospitalization (32).

Two previous clinical trials showed that ~1 g/day of n-3 PUFA supplementation did not affect the frailty score and incidence of frailty in ≥70-year-old French (33) and in ≥50-year-old Americans (34). Meta-analysis of clinical trials revealed that muscle mass increased in older adults supplemented >2 g/day of n-3 PUFA (15). In addition, a few clinical trials have shown that muscle function, such as handgrip strength (35), timed up and go test (36), and gait speed (37), had improved in older adults supplemented >2 g/day n-3 PUFA, suggesting that >2 g of n-3 PUFA might be required to improve the frailty status.

Otsuka et al. (38) reported that the intake of total seafood and n-3 PUFA did not significantly differ among the frailty transition groups of deterioration, persistence, and reversal in older Japanese adults in a 2-year follow-up. The present study demonstrated that the total seafood and fish intake did not differ significantly between the frailty transition groups. A meta-analysis of clinical trials also showed that nutritional supplements did not affect frailty status or frailty scores (39).

In the present study, shellfish intake was not significantly associated with the prevalence of frailty at baseline or at the 4-year follow-up. Shellfish contains lesser amount of EPA and DHA than fish (40). A meta-analysis of clinical studies reported that supplementation of EPA + DHA derived from fish oil significantly reduced blood levels of C-reactive protein, interleukin-6 (IL-6), and tumor necrosis factor-α (41). However, Fu et al. (42) showed that supplementation of lipid extract from mussel did not reduce blood levels of IL-6 in patients with rheumatoid arthritis.

The present study showed that the total seafood intake, including fish, was inversely associated with the prevalence of exhaustion at baseline and at the 4-year follow-up. Previously, we have also reported that the erythrocyte levels of DHA were negatively associated with the likelihood of exhaustion, as assessed using two questions from the CES-D scale in older Korean adults (5). In addition, an epidemiological study showed that depressive symptoms, assessed using the CES-D scale were negatively associated with the intake of seafood in Korean adults (43) and fish in Japanese adults (44).

Consistent with the observations of this present study, cross-sectional epidemiological studies showed that consumption of seafood was positively associated with gait speed in older Norwegian women (11) and ≥ 50-year-old Australians with T2DM (45). Gait speed was also positively associated with intake of n-3 PUFA in older Finnish women (46), and with EPA intake in older Australians with subjective memory complaints (47). Furthermore, plasma levels of n-3 PUFA were associated with faster gait speed in Italian women at a 3-year follow-up (48), and with lower risk of mobility disability, defined as having difficulty in walking or climbing steps, in older Icelandic women (49). Alhussain et al. (50) reported that gait speed increased significantly after consumption of fish during 10 weeks in older Saudi Arabian adults. A meta-analysis of clinical studies revealed that n-3 PUFA supplementation improved gait speed in older Americans and Europeans (15).

The present study showed that the intake of total seafood and fish was associated with handgrip strength at baseline and at the 4-year follow-up. Consumption of fish significantly increased handgrip strength in older Saudi Arabian adults (50). A meta-analysis of the clinical studies also showed that n-3 PUFA supplementation improved handgrip strength in older Americans and Europeans (14). Consistently, previous cross-sectional epidemiological studies have shown that handgrip strength was positively associated with the intake of fatty fish and oily fish in older adults from the UK (12, 13).

This was the first study to show that the intake of not only total seafood, but also fish, was inversely associated with the prevalence of frailty among older adults during a 4-year follow-up period. However, this study had several limitations. First, the power to detect the incidence of frailty was only 0.74, suggesting that the small sample size may have attenuated the strength of our results. Second, dietary data were obtained using two non-consecutive days of 24-h dietary recalls during two different seasons; however, this might not be sufficient to determine the participants’ usual diet. Third, our findings were obtained only from Korean ambulatory older adults, which may not be generalized for the older population.

## 5. Conclusion

The present study suggests that the total consumption of seafood, including fish, could be beneficial for preventing frailty in older Korean adults. Further population-based longitudinal studies are required to verify whether the marine fat composition and content are associated with the incidence of frailty.

## Data availability statement

The data analyzed in this study is subject to the following licenses/restrictions: the data are not publicly available due to privacy or ethical restrictions. Requests to access these datasets should be directed to mijiak@khu.ac.kr.

## Ethics statement

The studies involving humans were approved by the Institutional Review Boards (KHUH-2015-12-103-107 and HYUIRB-202303-016). The studies were conducted in accordance with the local legislation and institutional requirements. The participants provided their written informed consent to participate in this study.

## Author contributions

YP was responsible for study concept and design. CW and MK was responsible for acquisition of data. JA was responsible for analysis and drafting of the manuscript. CW, MK, and YP were responsible for critical revision of the manuscript for important intellectual content. All the authors have read and agreed to the published version of the manuscript.

## Funding

This study was supported by a grant of the Korea Health Technology R&D Project through the Korean Health Industry Development Institute (KHIDI) funded by the Ministry of Health and Welfare, Republic of Korea (grant number HI15C3153), and the National Research Foundation of Korea (NRF) grant funded by the Korea government (grant number NRF-2021R1A2B02002208).

## Conflict of interest

The authors declare that the research was conducted in the absence of any commercial or financial relationships that could be construed as a potential conflict of interest.

## Publisher’s note

All claims expressed in this article are solely those of the authors and do not necessarily represent those of their affiliated organizations, or those of the publisher, the editors and the reviewers. Any product that may be evaluated in this article, or claim that may be made by its manufacturer, is not guaranteed or endorsed by the publisher.
