# Supplementary material for: Association between fish intake and prevalence of frailty in community-dwelling older adults after 4-year follow-up: the Korean frailty and aging cohort study
Source: Front Nutr. 2023 Aug 29;10:1247594. doi: 10.3389/fnut.2023.1247594 (PMC10497173; doi:10.3389/fnut.2023.1247594)
Supplement: Supplementary file 1 [file Table_1.DOCX]

Supplementary Material

Association Between Fish Intake and Prevalence of Frailty in Community-dwelling Older Adults After 4-year Follow-up: the Korean Frailty and Aging Cohort Study

**Jeonghwan Ahn^1^, Miji Kim, PhD^2^, Chang Won Won, MD, PhD^3^, Yongsoon Park, PhD1***

^1^ Department of Food and Nutrition, Hanyang University, Seoul, Republic of Korea

^2^ Department of Biomedical Science and Technology, College of Medicine, East-West Medical Research Institute, Kyung Hee University, Seoul, Republic of Korea

^3^ Department of Family Medicine, College of Medicine, College of Medicine, Kyung Hee University, Seoul, Republic of Korea

*** Correspondence:**Yongsoon Park, PhD
yongsoon@hanyang.ac.kr

# Supplementary Table

**Supplementary Table 1.** Logistic regression of seafood intake among non-frail participants at the baseline for the incidence of frailty after 4-year follow-up

|  | Tertiles of dietary intake (n =563) | | | *p* for trend | Dietary intake Continuous | |
| --- | --- | --- | --- | --- | --- | --- |
|  | T1 | T2 | T3 |  | OR (95% CI) | *p*-value |
| Total seafood, g/day | ≤ 20.00 | 20.00 < to ≤ 60.25 | > 60.25 |  |  |  |
| Frail, n (%) | 23 (12.3) | 18 (9.6) | 13 (6.9) |  |  |  |
| Adjusted OR (95% CI) | 1.0 (ref) | 0.809 (0.395-1.657) | 0.560 (0.254-1.236) | 0.15 | 0.995 (0.989-1.002) | 0.19 |
| Fish, g/day | ≤ 9.50 | 9.50 < to ≤ 46.25 | > 46.25 |  |  |  |
| Frail, n (%) | 21 (11.2) | 19 (10.2) | 14 (7.4) |  |  |  |
| Adjusted OR (95% CI) | 1.0 (ref) | 0.899 (0.437-1.852) | 0.673 (0.307-1.476) | 0.32 | 0.995 (0.987-1.003) | 0.19 |
| Shellfish, g/day | 0 | 0 < to ≤ 7.50 | > 7.50 |  |  |  |
| Frail, n (%) | 28 (9.4) | 12 (14.1) | 14 (7.8) |  |  |  |
| Adjusted OR (95% CI) | 1.0 (ref) | 2.289 (1.032-5.079) | 1.264 (0.608-2.627) | 0.78 | 0.997 (0.983-1.011) | 0.65 |

OR, odds ratio; CI, confidence interval; ref; reference. Estimate of p for linear trends was based on linear scores derived from the medians of tertiles of seafood intake among all participants. Adjusted OR and 95% CI were analyzed using logistic regression analysis after adjusting for sex, age, medications, cognitive impairment, fall experience, body mass index, and nutritional status. Estimate of p-value were derived from the continuous variables of dietary intake among all participants after adjusting for sex, age, medications, cognitive impairment, fall experience, body mass index, and nutritional status.
